# Zilucoplan, a macrocyclic peptide inhibitor of human complement component 5, uses a dual mode of action to prevent terminal complement pathway activation

**DOI:** 10.3389/fimmu.2023.1213920

**Published:** 2023-08-09

**Authors:** Guo-Qing Tang, Yalan Tang, Ketki Dhamnaskar, Michelle D. Hoarty, Rohit Vyasamneni, Douangsone D. Vadysirisack, Zhong Ma, Nanqun Zhu, Jian-Guo Wang, Charlie Bu, Bestine Cong, Elizabeth Palmer, Petra W. Duda, Camil Sayegh, Alonso Ricardo

**Affiliations:** ^1^ UCB Pharma, Cambridge, MA, United States; ^2^ Ra Pharmaceuticals, Cambridge, MA, United States; ^3^ UCB Pharma/Ra Pharmaceuticals, Cambridge, MA, United States

**Keywords:** complement activation, C5 cleavage, C5 R885 variants, C5b6, MAC formation, macrocyclic peptide inhibitor, RBC hemolysis, permeability

## Abstract

**Introduction:**

The complement system is a key component of the innate immune system, and its aberrant activation underlies the pathophysiology of various diseases. Zilucoplan is a macrocyclic peptide that binds and inhibits the cleavage/activation of human complement component 5 (C5). We present *in vitro* and *ex vivo* data on the mechanism of action of zilucoplan for the inhibition of C5 activation, including two clinically relevant C5 polymorphisms at R885.

**Methods:**

The interaction of zilucoplan with C5, including for clinical C5 R885 variants, was investigated using surface plasmon resonance (SPR), hemolysis assays, and ELISA. The interference of C5b6 formation by zilucoplan was investigated by native gel analysis and hemolysis assay. The permeability of zilucoplan in a reconstituted basement membrane was assessed by the partition of zilucoplan on Matrigel-coated transwell chambers.

**Results:**

Zilucoplan specifically bound human complement C5 with high affinity, competitively inhibited the binding of C5 to C3b, and blocked C5 cleavage by C5 convertases and the assembly of the cytolytic membrane attack complex (MAC, or C5b9). Zilucoplan fully prevented the *in vitro* activation of C5 clinical variants at R885 that have been previously reported to respond poorly to eculizumab treatment. Zilucoplan was further demonstrated to interfere with the formation of C5b6 and inhibit red blood cell (RBC) hemolysis induced by plasmin-mediated non-canonical C5 activation. Zilucoplan demonstrated greater permeability than a monoclonal C5 antibody in a reconstituted basement membrane model, providing a rationale for the rapid onset of action of zilucoplan observed in clinical studies.

**Conclusion:**

Our findings demonstrate that zilucoplan uses a dual mode of action to potently inhibit the activation of C5 and terminal complement pathway including wild-type and clinical R885 variants that do not respond to eculizumab treatment. These data may be relevant to the clinically demonstrated benefits of zilucoplan.

## 1 Introduction

The complement system is part of the innate immune system and plays a critical role in the destruction of pathogenic bacteria. Three complement pathways are known: the classical, lectin, and alternative pathways (1). The classical pathway (CP) is activated by surface-bound immunoglobulins, and the lectin pathway (LP) is activated by mannose-binding lectin recognizing sugar residues on bacterial cell walls. The alternative pathway (AP) remains continuously active at low levels in the absence of any specific stimuli and is controlled by endogenous regulators.

Irrespective of the initiating event, activation pathways of the complement system converge at the point of cleavage of the complement protein C5 (~190 kDa) into C5a (~10 kDa) and C5b (~180 kDa). C5a is a potent anaphylatoxin, and C5b binding to C6 triggers the terminal complement cascade (TCC), resulting in the membrane attack complex (MAC) formation by seeding the deposition of complement proteins C6, C7, C8, and C9 (2). The MAC is a hydrophilic pore that spans the cell membrane and promotes an influx of water and ions, thereby causing osmotic lysis of the targeted cell (3).

In autoantibody-driven autoimmune diseases, autoantibody formation and recognition of an antigen on self-cells may trigger activation of the classical complement pathway, leading to MAC deposition and localized injury specific to tissues expressing the autoantigen (4). In recent years, several therapeutics targeting complement component C5 have been assessed in complement-mediated diseases such as myasthenia gravis (MG) (5), neuromyelitis optica spectrum disorders (NMOSDs) (6), paroxysmal nocturnal hemoglobinuria (PNH) (7), and atypical hemolytic uremic syndrome (aHUS) (8). Additional therapeutics targeting C5 are currently in clinical development for various indications (9).

Zilucoplan is a fully synthetic macrocyclic peptide developed through a targeted screen using the innovative extreme diversity mRNA display platform (10–12). It is composed of a 15-amino acid macrocyclic peptide, including four unnatural amino acids, designed to inhibit TCC activation (13). The addition of a C16 lipid via a short monodisperse polyethylene glycol (PEG) linker provides a pharmacokinetic (PK) profile consistent with daily dosing in humans. Zilucoplan is self-administered once daily as a subcutaneous (SC) injection taking a few seconds (14). Pharmacologically, zilucoplan has demonstrated dose-dependent inhibition of complement activation in humans, as measured *ex vivo* by a red blood cell (RBC) lysis assay (15). Here, we provide additional information on the interaction between zilucoplan and complement C5 and the ability of zilucoplan to prevent the activation of C5 in both wild-type and two clinically relevant variants (R885C/H) that have been shown to be resistant to eculizumab treatment in PNH patients carrying such polymorphisms (16). Our data demonstrate that zilucoplan inhibits the MAC formation via a dual mechanism of action: preventing C5 cleavage by C5 convertases and competitively inhibiting the formation of C5b6 such as arising from non-canonical C5 activation (17, 18).

## 2 Materials and methods

### 2.1 Ethics statement

This study used pooled human or animal sera. No biospecimen samples from patients and no human or animal subjects were involved in the study.

### 2.2 Reagents

Antibody-sensitized sheep erythrocytes (EA), rabbit erythrocytes, normal human serum (NHS), C5-depleted or C9-depleted human serum, human complement proteins (including C5, C6, C7, C8, C9, and C5b6), and gelatin veronal buffer (GVB) and gelatin veronal buffer with calcium and magnesium (GVB++) were purchased from Complement Technology, Inc. (Tyler, TX, USA). Human C9 protein was conjugated with Alexa Fluor 647 dye using a labeling kit (Invitrogen, Carlsbad, CA, USA). Zilucoplan was prepared internally (UCB Ra Pharmaceuticals, Cambridge, MA, USA). Eculizumab biosimilar was purchased from Syd Labs Inc. (Hopkinton, MA, USA). Labeling of eculizumab biosimilar with Alexa Fluor™ 488 NHS Ester (AF488) (Thermo Fisher, Waltham, MA, USA) was conducted using a labeling kit and following the vendor’s protocol (**Supplementary Material**). Fluorescein isothiocyanate (FITC)-labeled dextran (molecular weight approximately 4,000 and 150,000 Da; FITC-dextran 4 kDa and FITC-dextran 150 kDa, respectively) was from Chondrex (Woodinville, WA, USA).

Animal sera used in cross-species activity assay included rat serum (Complement Technology) and sera of baboon, beagle, chimpanzee, cynomolgus monkey, guinea pig, minipig, mouse, pig, rhesus monkey, and rabbit (all from BioIVT, Hicksville, NY, USA).

### 2.3 Hemolysis assay

Inhibition of the complement classical pathway activation by zilucoplan was determined in a hemolysis assay using EA exposed to 1% NHS in GVB++ buffer. Briefly, serial dilutions of zilucoplan were mixed with NHS, EA, and GVB++ in 96-well plates at 37°C. The samples were centrifuged to pellet the remaining intact erythrocytes, and supernatants were collected. Hemoglobin release from erythrocyte lysis was detected by measuring the optical density (OD) of the supernatant at 412 nm using a microplate reader (SpectraMax M4 from Molecular Devices, or Tecan SPARK, from Tecan, Männedorf, Switzerland). Percentages of hemolysis were plotted against inhibitor concentration and fitted to a standard four-parameter dose–response inhibition function (GraphPad Prism) to calculate IC_50_ (50% inhibition) and IC_90_ (90% inhibition) values.

### 2.4 C5a and sC5b9 levels by ELISA

C5a and sC5b9 (soluble MAC) levels in supernatants from the CP-mediated hemolysis assay (Section 2.3) were quantified using Microvue™ Complement EIA kits (Quidel Corporation, San Diego, CA, USA). Of the supernatant in the sample diluent, the C5a ELISA was performed at 1:5 dilution, and the sC5b9 ELISA was performed at 1:2 dilution. Data were converted to a percentage of C5a or sC5b9 production inhibition and fitted to a standard four-parameter dose–response inhibition function (GraphPad Prism) to calculate IC_50_ values.

### 2.5 ABO incompatibility-based hemolysis

Inhibition of the complement activation in a high blood concentration by zilucoplan was further characterized using an ABO incompatibility RBC hemolytic assay. Of human RBCs from blood group B+ donors (BioIVT), 2.5% were exposed to incompatible 86.5% human serum from blood group A+ donors (BioIVT) in Alsever’s solution without or with zilucoplan. Serial dilutions of zilucoplan were mixed with RBC and human serum in 96-well plates at 37°C. The samples were handled and analyzed as described for the hemolysis assay.

### 2.6 Complement AP, CP, and LP activation by Wieslab ELISA

Inhibition of the activation of complement pathways (AP, CP, and LP) by zilucoplan was assessed by using Wieslab Complement System ELISA kits (Euro Diagnostica, Malmö, Sweden), which measured the amount of C5b9 formed on the plate surface. Serial dilutions of zilucoplan were combined with 1% NHS for the CP strips, 2% NHS for the LP strips, and 5.5% NHS for the AP strips. Samples were added to the strips in duplicate and incubated for 1 h at 37°C. The strips were processed by washing, antibody conjugation, and substrate incubation at room temperature, according to the manufacturer’s protocol. OD at 405 nm was read using a SpectraMax M4 microplate reader. Data were converted to a percentage of MAC production inhibition and fitted to a standard four-parameter dose–response inhibition function (GraphPad) to calculate IC_50_ values.

### 2.7 Cross-species hemolysis activity

A CH_50_ test that measures the activity of complement CP activation in hemolysis was conducted for the serum or plasma of each animal species to be examined. Antibody-sensitized sheep erythrocytes were then exposed to serum or plasma from each species at the experimentally determined CH_50_ in the presence of varying concentrations of zilucoplan. The samples were centrifuged to pellet the remaining intact erythrocytes. Supernatants were collected to measure the absorbance at 412 nm for released hemoglobin as a result of hemolysis. Data analysis was performed as described in Section 2.3.

### 2.8 C3b and MAC deposition on human umbilical vein endothelial cells

Human umbilical vein endothelial cells (HUVECs) were cultured and seeded in 96-well plates. Cell surface depositions of complement C3b and MAC (C5b9) were triggered by the activation of the complement CP pathway using an anti-CD59 antibody (MEM-43, mouse IgG2a) and human serum in the presence or absence of zilucoplan. Detailed methods for cell cultures, complement activation, and immunostaining are described in the **Supplementary Material**.

### 2.9 Native gel analysis of C5b6 complex

Human sera isolated C5b6 complex (Complement Technology) at a concentration of 0.2 mg/ml (~700 nM) was incubated with serial dilutions of zilucoplan at 37°C for 1 h and then mixed with 4× sample loading buffer (G-250). Sample mixtures were loaded to a precast 4%–16% Invitrogen NativePAGE Bis-Tris gel (Thermo Fisher), and the gel ran at 150 V for ~2 h at room temperature. Proteins in gel were visualized with Coomassie stains.

### 2.10 Plasmin-mediated C5 activation and hemolysis

Plasmin-mediated C5 activation was performed by adding 100 nM of plasmin (Sigma-Aldrich Corp., St. Louis, MO, USA) with or without zilucoplan at varying concentrations to a mixture of equimolar purified human C5 and C6 (400 nM each) and incubation at 37°C for 1 h. The cleavage reaction was stopped by adding 10 µM of proteinase inhibitor aprotinin (Sigma). The reaction solution was incubated with EA (0.5×) (Complement Technology) in GVB++ buffer at 37°C for 5 min, followed by the addition of purified human C7 (40 nM), C8 (40 nM), and C9 (80 nM), and further incubation at 37°C for 30 min. The assay plate was centrifuged to pellet the cells, and the supernatant was transferred to measure the absorbance at 412 nm. Data analysis was performed as described in Section 2.3.

### 2.11 Complement activation by C5 R885 variants

Recombinant human C5 R885 variants (rhC5 R885C and rhC5 R885H) were prepared in-house (**Supplementary Material**). Their activities on complement activation were examined by the CH_50_ test using 1.5% C5-depleted human sera supplemented with C5 R885 variants. Inhibition by zilucoplan or eculizumab biosimilar was examined using the hemolysis of EA exposed to 1.5% C5-depleted human serum supplemented with external C5 (1.5 nM of C5 wt purified from human sera; or recombinant C5 wt, rhC5 wt, 20 nM of rhC5 R885C; or 5 nM of rhC5 R885H). Other procedures and data analysis are described in Section 2.3.

### 2.12 Surface plasmon resonance assay

Surface plasmon resonance (SPR) assay was performed on a Bio-Rad ProteOn XPR36 (Bio-Rad, Hercules, CA, USA) or a Biacore 8K (formerly GE Healthcare, now part of Cytiva, Marlborough, MA, USA). Human complement C5 proteins (wt or R885 variants) were immobilized on a Bio-Rad GLH sensor chip docked in ProteOn or on a CM5 sensor chip docked in Biacore 8K, followed by flowing of zilucoplan or eculizumab biosimilar at varying concentrations in 1× HEPES buffer (pH 7.4, 150 mM of NaCl, 1 mM of MgCl_2_, 0.005% surfactant P-20, and 1% dimethyl sulfoxide (DMSO) or 1× phosphate-buffered saline (PBS) buffer (pH 7.4, 0.005% P-20, and 1% DMSO). The resulting SPR sensorgrams were recorded and analyzed using the software provided by the vendors to extract the association and dissociation rate constants (k_a_ and k_d_) and the binding affinity (KD).

For the binding of C5 and C3b, human C3b (Complement Technology) was site-specifically biotinylated via the thioester using Thermo Fisher EZ-link™ maleimide-PEG2-biotin. Biotinylated C3b was immobilized on a Bio-Rad neutravidin sensor chip. C5 in the absence and presence of zilucoplan in 1×HEPES buffer pH 7.4 was flowed over the immobilized C3b, and the resulting SPR signals were recorded using a Bio-Rad ProteOn XPR36.

### 2.13 Permeability assay on reconstituted basement membrane

Transwell chambers were coated with Matrigel (both purchased from Corning Life Sciences, Tewksbury, MA, USA) at 37°C overnight. Zilucoplan, eculizumab biosimilar labeled with Alexa Fluor™ 488 (AF488), FITC-dextran 4 kDa, and FITC-dextran 150 kDa in PBS solution were separately added to transwell chamber wells at 37°C. Aliquots were collected from both upper and lower chambers at prespecified time points and transferred to high-performance liquid chromatography (HPLC) vials (zilucoplan) or microplates (AF488-eculizumab and FITC-dextran) for analysis. Zilucoplan was detected by HPLC-MS analysis; eculizumab biosimilar and dextran were detected by measuring the fluorescence intensity (excitation 490 nm and emission 520 nm) using a Tecan Spark microplate reader. Their respective ratios of the lower chamber relative to upper chamber quantities were calculated (**Supplementary Material**).

## 3 Results

### 3.1 Zilucoplan binds to human C5 and blocks the terminal complement pathway activation

**Figure 1:**
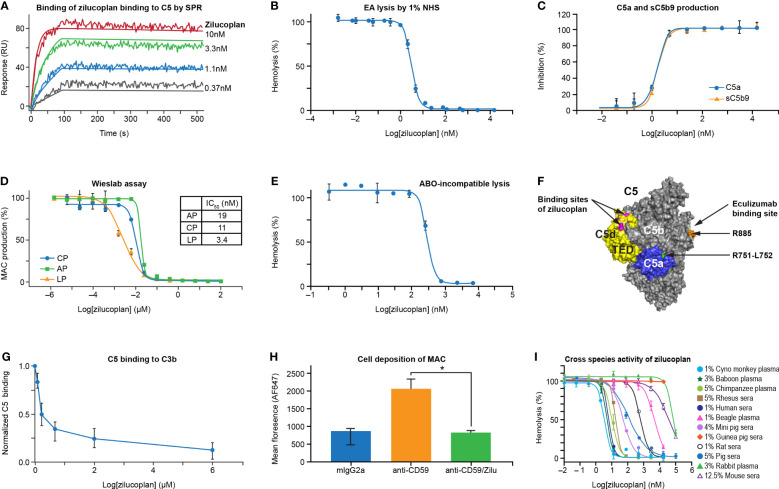
Zilucoplan binds human C5 and blocks complement-mediated hemolysis *in vitro*. **(A)** SPR assay shows the binding of zilucoplan with human C5 (SPR sensorgram as fluctuating lines and fitting as linear lines at each concentration). **(B)** Zilucoplan inhibits the lysis of antibody-sensitized sheep red blood cells exposed to 1% NHS. **(C)** ELISA measurement of C5a and sC5b9 in the supernatant of RBC hemolysis described in panel **(B, D)** Wieslab assay to measure the production of MAC in CP, LP, and AP activation. **(E)** Inhibition of ABO incompatibility-based hemolysis by zilucoplan. **(F)** Graphical representation (generated using PyMOL 2.4.0 based on PDB 5I5K) of the binding site of a peptide analog of zilucoplan (in red, unpublished data) on C5 TED (in yellow), R885 (in orange) as part of eculizumab epitopes on C5b (in gray and including TED), and the cleavage site at R751 (in green) within C5a (in blue). **(G)** Zilucoplan inhibition of C5 (200 nM) binding to surface C3b measured by SPR, shown as mean ± SD (normalized to the signal at zero zilucoplan, N = 5). **(H)** Zilucoplan inhibited the deposition of MAC on HUVECs. *, p value <0.05. Fluorescence intensity was shown as mean ± SD (n = 3 wells in each group). Statistical significance was analyzed by ANOVA (Kruskal–Wallis test) using GraphPad software (Version 9.2.0). **(I)** The activity of hemolysis inhibition of zilucoplan is potent in non-human primates and drastically reduced in guinea peak, rats, and mice. SPR, surface plasmon resonance; NHS, normal human serum; RBC, red blood cell; MAC, membrane attack complex; CP, classical pathway; LP, lectin pathway; AP, alternative pathway; TED, thioester-like domain; HUVECs, human umbilical vein endothelial cells.

The binding kinetics and affinity of zilucoplan to human C5 were demonstrated by SPR measurement using a ProteOn XPR36 on which C5 was immobilized on the surface via amine coupling. **Figure 1A** shows a representative sensorgram of simultaneous titration of zilucoplan to surface-immobilized C5 at 25°C measured on a Bio-Rad ProteOn XPR-36. Data fitting using a 1:1 kinetic model revealed an association rate constant (k_a_) of 6.3 × 10^5^ M^−1^ s^−1^ and a dissociation rate constant (k_d_) of 2.1 × 10^−4^ s^−1^, resulting in a high binding affinity (KD) of 0.43 nM (averaged over four independent measurements) for the binding of zilucoplan and C5. Zilucoplan bound neither complement components such as C3 or C4 that share a highly similar three-dimensional structure with C5 nor C6 or C7 that associate with C5b to initiate the MAC formation (2) (data not shown).

The ability of zilucoplan to block the activation of TCC was assessed by lysis of antibody-sensitized sheep erythrocytes (EA) exposed to 1% NHS in the absence and presence of serial concentrations of zilucoplan (**Figure 1B**). In the complement CP-mediated hemolytic assay, zilucoplan displayed dose-dependent inhibition of the lysis to completion with an IC_50_ of 3.2 nM. Additionally, two soluble terminal activation products in the supernatant of the hemolysis reaction, C5a and sC5b9, were measured by ELISA. Zilucoplan demonstrated dose-dependent inhibition of C5a and sC5b9 production with potency (IC_50 _= 1.6 nM for C5a and 1.7 nM for sC5b9) similar to those measured from the lysis (**Figure 1C**), consistent with a mode of action by which zilucoplan binds C5 and blocks its cleavage into C5a and C5b by C5 convertases. As expected for a C5 inhibitor, zilucoplan potently inhibited the activation of all three complement pathways as demonstrated by a Wieslab assay of the MAC deposition (**Figure 1D**). Further, in an ABO incompatibility-based human RBC hemolytic assay using a high level of human blood, zilucoplan demonstrated a steep dose–response curve of inhibition with IC_50_ and IC_90_ values of 284 and 563 nM, respectively (**Figure 1E**), indicating stoichiometric binding and inhibition of C5 by zilucoplan.

It is not obvious how zilucoplan, with its relatively small size and binding site on the C5d/thioester-like domain (TED) distal to the convertase cleavage site at Arg751-Leu752, achieves the potent inhibition of C5 cleavage by C5 convertases (**Figure 1F**). Plausible mechanisms include disturbance of the binding of C5 with C5 convertases or zilucoplan binding-induced conformational changes of C5 such as in the vicinity of the cleavage site. It has been shown that C3b in C5 convertases is required to bind C5 to facilitate C5 cleavage, and this binding can be disrupted by C5 inhibitors (19–21). To assess whether zilucoplan uses a similar mechanism, biotinylated C3b was captured on a neutravidin surface, and its binding of C5 was evaluated by SPR in the absence and presence of zilucoplan. Indeed, the binding of C5 to surface C3b was blocked by zilucoplan (**Figure 1G**), suggesting that zilucoplan may compete with the C3b of C5 convertases for the binding of C5.

Inhibition of complement fixation on cells other than RBCs by zilucoplan was investigated in an *in vitro* cell-based assay using primary HUVECs. Cell surface-bound anti-CD59 antibody (MEM-43, mouse IgG2a) triggered activation of the complement classical pathway, resulting in the deposition of both C3b fragments and MAC on the cell membrane as demonstrated by immunofluorescence images (**Supplementary Figure 1**). Incubation with zilucoplan caused a complete blockade of the deposition of MAC, but not C3b fragments, which are activation products upstream of C5 (**Figure 1H** and **Supplementary Figure 1**). No change of endothelial cell marker PECAM-1/CD31 expression was observed by using any of the antibody reagents (isotype, CD59, and CD59+zilucoplan). Collectively, these data on direct binding, hemolytic lysis, and complement fixation demonstrate that zilucoplan binds C5 and prevents C5 cleavage and terminal complex formation with high potency.

Zilucoplan demonstrated potent inhibition of complement-induced RBC lysis by serum and/or plasma of non-human primates (cynomolgus monkey, baboon, chimpanzee, and rhesus monkey), with IC_50_ values under 18 nM for these species (**Figure 1I**). Reduced activity was observed in mini pig, pig, and rat sera, with IC_50_ values ranging from 50 to 609 nM. Only minimal activity (IC_50_ > 4 µM) was observed in other species including beagle, mouse, rabbit, and guinea pig.

### 3.2 Zilucoplan inhibits activation of eculizumab-resistant C5 R885 mutants

**Figure 2:**
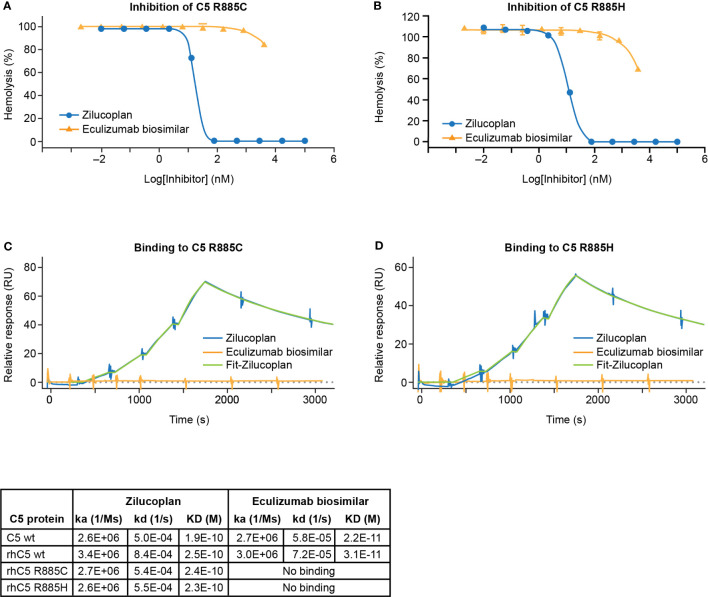
Zilucoplan blocks complement activation by C5 variants R885C/H. (A, B) Inhibition of the lysis of EA upon the activation of rhC5 R885C **(A)** or R885H **(B)** was tested for zilucoplan (circle) or eculizumab biosimilar (triangle). (C, D) SPR measurement (in a single cycle kinetics mode using Biacore 8K) of zilucoplan or eculizumab biosimilar to rhC5 R885 **(C)** or R885H **(D)** at 37°C. Surface densities of C5 proteins for zilucoplan testing: sera C5 wt-5970RU, rhC5 wt-6874RU, rhC5 R885C-6496RU, and rhC5 R885H-6145RU. Zilucoplan concentrations used: 0, 0.31, 0.62, 1.25, and 2.5 nM. Surface densities of C5 proteins for eculizumab testing: sera C5 wt-715RU, rhC5 wt-840RU, rhC5 R885C-731RU, and rhC5 R885H-767RU. Eculizumab biosimilar concentrations used: 0, 0.31, 0.62, 1.25, and 2.5 nM on C5 wt and 0, 11, 33, 100, and 300 nM on C5-R885C/H. Binding kinetics and affinity for zilucoplan and eculizumab biosimilar on C5 wt from human sera or recombinant forms (rhC5 wt, R885C, and R885H) from two independent measurements are also summarized in tabular format. For the clarity of figures, only the SPR sensorgrams for the mutants are shown. SPR, surface plasmon resonance.

The ability of zilucoplan to inhibit the activation of human C5 variants at R885 (R885C or R885H) was assessed by the lysis of EA using 1.5% C5-depleted human serum supplemented with C5 proteins (wild type or mutants). Recombinant protein carrying either R885C or R885H mutation (rC5 R885C/H) preserved the capacity of complement activation as demonstrated by the induction of complete lysis of EA (**Supplementary Figure 2**). In the assay, both variants retained the complement hemolytic activity despite larger values of CH_50_ of 5.2 or 1.1 nM respectively for rhC5 R885C or rhC5 R885H than those of C5 wt (0.2–0.3 nM), consistent with reduced activity of complement activation by such polymorphisms as reported (22). With the use of 20 nM of C5 rhC5 R885C or 5 nM of rhC5 R885H that led to the full lysis of EA, zilucoplan was shown to inhibit the lysis with IC_50_ of 18 or 11 nM, respectively, and accomplish full inhibition at 80 nM and above of zilucoplan (**Figures 2A, B**). In contrast, EA lysis was barely (R885C) or only slightly (R885H) inhibited by eculizumab biosimilar even at a high concentration of 4 µM. When the binding of zilucoplan or eculizumab similar to C5 (wt and variants) was measured by SPR (in a single cycle kinetics mode, **Figures 2C, D**), zilucoplan showed almost identical binding kinetics and affinity between C5 wt and R885 variants (**Figure 2** table). In contrast, no binding was observed for eculizumab biosimilar for C5 R885 variants despite >100-fold higher concentrations of eculizumab biosimilar used on rhC5 R885C/H surface than on C5 wt surface (300 versus 2.5 nM). These functional and binding results are consistent with a previous report of eculizumab failing to bind and neutralize these human C5 variants (16). The difference in inhibition of C5 R885 C/H mutants between zilucoplan and eculizumab is consistent with their distinct binding sites on C5, with R885 being within an epitope of eculizumab spanning K879 to R885 (23) while remote from the C5d/TED where zilucoplan binds (**Figure 1F**).

### 3.3 Zilucoplan interferes with the formation of C5b6

**Figure 3:**
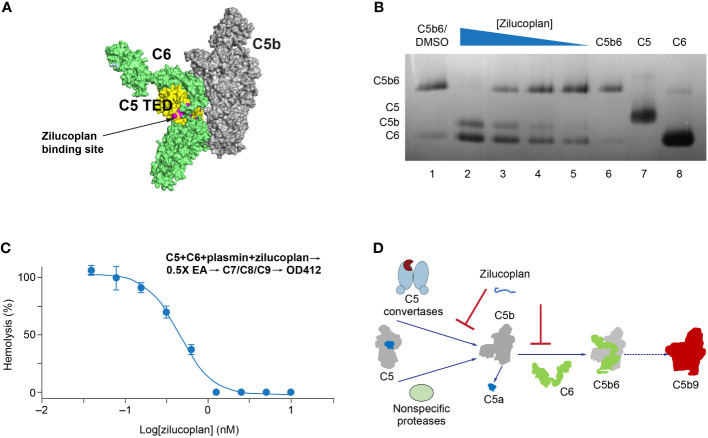
Zilucoplan destabilizes C5b6 and inhibits C5b6-mediated hemolysis lysis. **(A)** Schematic depiction (using PyMOL 2.4.0 based on PDB 4a5w) of the C5b6 complex. C5b is in gray except for the C5 TED in yellow, proposed binding sites for zilucoplan peptidyl portion based on a peptide analog (unpublished data) in pink, and C6 in lime. **(B)** Native gel analysis of the C5b6 complex in the presence and/or absence of zilucoplan. Lanes 1 and 6: C5b6 incubated at 37°C/h with (Lane 1) or without (Lane 6) 1% DMSO. Lanes 2–6: C5b6 after incubation with various concentrations of zilucoplan (in 1% DMSO) at 37°C for 1 h prior to gel loading. Lanes 7 and 8: C5 and C6 alone. **(C)** Zilucoplan inhibited the plasmin-mediated lysis of EA. Plasmin without or with increasing concentrations of zilucoplan was added to a mixture of C5 and C6 and incubated for 1 h. The plasmin reaction solution was then added to EA before mixing with purified C7, C8, and C9 to proceed with the lysis. More details are described in the Materials and Methods. Note that no sera were used to exclude any contribution by C3/C5 convertases. **(D)** A schematic diagram of the dual mechanism of action for zilucoplan: zilucoplan binds C5 (gray/dark blue) to prevent C5 cleavage into C5a (dark blue) and C5b (gray) by C5 convertases (light blue) and remains on C5b to prevent the formation of C5b6 (C6 in green) mediated by nonspecific proteases, both contributing to the blockade of MAC formation (red). TED, thioester-like domain; DMSO, dimethyl sulfoxide.

Upon C5 cleavage, the nascent and labile C5b fragment is stabilized by binding to C6 and forms a highly stable C5b6 complex that initiates the assembly of MAC (24, 25). If zilucoplan binds the C5b fragment in a similar way and with a similar affinity to its binding with the full-length C5 (**Figure 3A**), it may compete with C6 and disturb the formation or the stability of C5b6. To test this, we used native gel electrophoresis to analyze the stability of a preformed C5b6 complex with zilucoplan preincubation (**Figure 3B**). C5b6 purified from human serum incubated at 37°C for 1 h prior to gel electrophoresis ran as a major band at the top (**Figure 3B**, Lanes 1 and 6). Preincubating the C5b6 complex with increasing concentrations of zilucoplan from 0.37 to 10 µM (threefold increase) led to the gradual disappearance of the C5b6 band and the appearance of two new lower bands (Lanes 2–5 in **Figure 3B**) corresponding to C5b (Lane 7) and C6 (Lane 8), consistent with the dissociation of C5b6 possibly by competitive binding of zilucoplan to C5b and displacing C6 from the complex.

To further demonstrate that zilucoplan may compete with C6 for nascent C5b and thus inhibit the terminal complement pathway, a protease-mediated extrinsic cleavage pathway for C5 was used. Plasmin is one of such non-specific proteases known to cleave C5 and form MAC to lyse RBC (26). Titrating zilucoplan simultaneously with plasmin to a mixture of C5 and C6 at a physiological concentration (400 nM each) did not prevent the cleavage of C5 into several fragments including ones with similar sizes as C5a and C5b (data not shown). Mixing the zilucoplan-absent cleavage solution with EA and other purified complement components C7, C8, and C9 resulted in the lysis of EA (**Figure 3C**), suggesting the formation of cytolytic MAC through plasmin-mediated extrinsic pathway as reported (17, 18). However, the lysis of EA was fully inhibited when increasing concentrations of zilucoplan were added together with plasmin (**Figure 3C**). Zilucoplan inhibited the lysis of EA with an IC_50_ of 450 nM. Taken together with the data of C5b6 dissociation by zilucoplan, the blockade of plasmin-mediated RBC lysis can be interpreted with a simple model by which zilucoplan binds nascent C5b and inhibits the formation of C5b6. Zilucoplan appears to exert a dual mechanism of action to inhibit MAC formation through 1) binding to C5 to block the accessibility of C5 convertases, thus preventing C5 cleavage, and 2) competition with C6 for the binding to nascent C5b including generated by non-specific proteases such as plasmin (**Figure 3D**).

### 3.4 Zilucoplan shows greater *in vitro* permeability than eculizumab biosimilar

**Figure 4:**
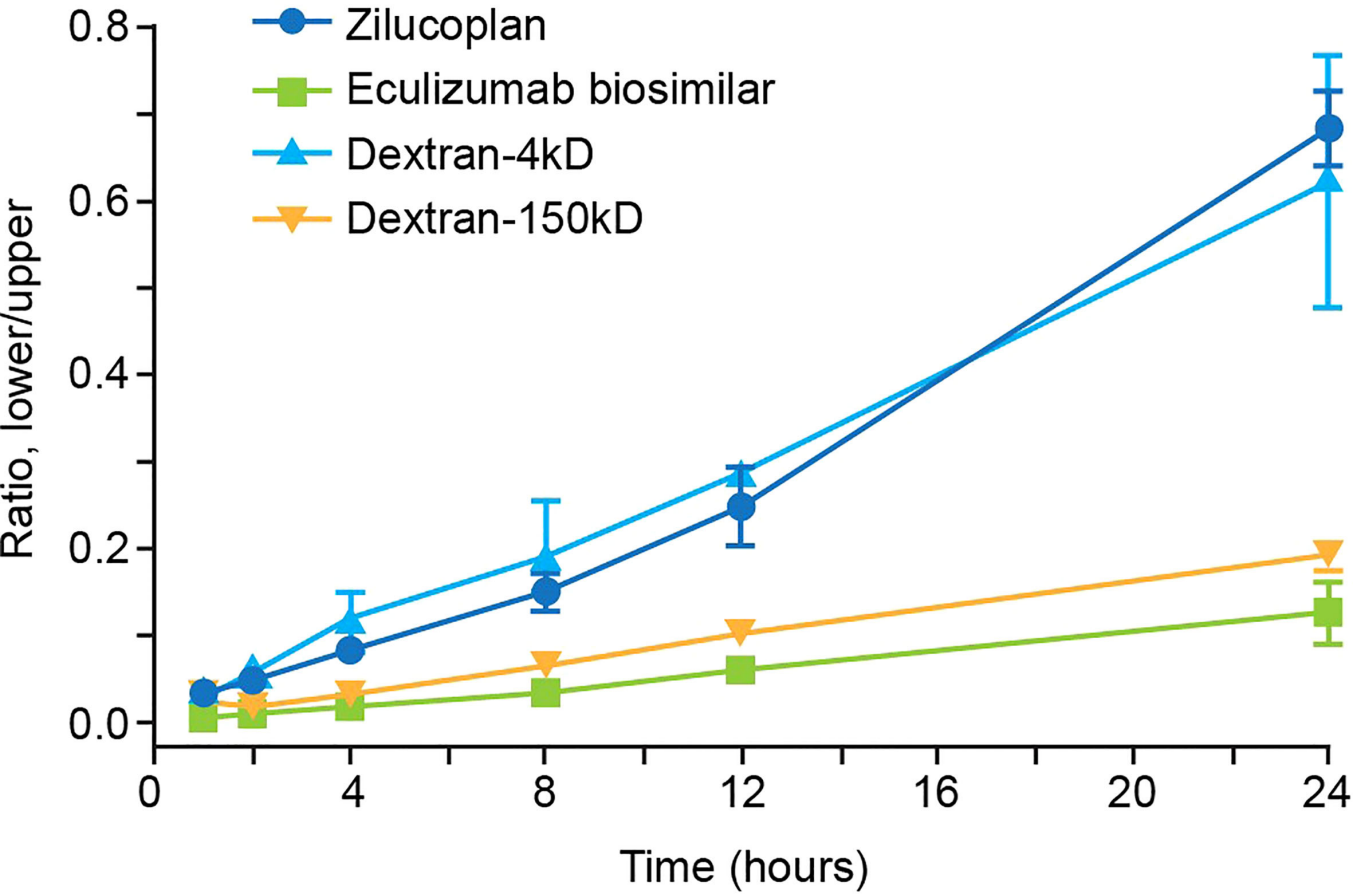
Analysis of the permeability of zilucoplan and eculizumab biosimilar. In a Matrigel-coated transwell assay, the concentrations of zilucoplan were measured by HPLC-MS to calculate the ratio between the lower and upper chambers. Fluorescence intensities of AF488-labeled eculizumab biosimilar or FITC-dextran (4 or 150 kDa) were measured in each chamber separately to calculate their respective ratios. Error bars represent ranges based on two or more independent plates with duplicates in each plate. Eculizumab biosimilar labeled with fluorophore AF488; dextran-4 kDa and dextran-150 kDa, FITC-dextran 4 kDa, or FITC-dextran 150 kDa. HPLC-MS, high-performance liquid chromatography–mass spectrometry; FITC, fluorescein isothiocyanate.

It is important for therapeutic molecules (including antibodies, peptides, and small molecules) to penetrate tissues freely and deeply following extravasation to effectively and selectively bind to target ligands that may be expressed inside the tissue. Permeability into tissue can be evaluated in an *in vitro* Matrigel model of a reconstituted basement membrane (27). In this assay, the efficiency of penetration of test molecules from the upper to the lower chambers, both coated with Matrigel, was assessed by the ratios between their concentrations measured in the lower chamber and in the upper chamber. As shown in **Figure 4**, the ratio of zilucoplan determined by liquid chromatography–mass spectrometry (LC-MS) increased approximately linearly with the incubation time, reaching approximately 0.68 in 24 h. The concentration of FITC-conjugated dextran 4 kDa, with its average molecular weight (4 kDa) close to zilucoplan (3.6 kDa), was evaluated by the fluorescence intensity of FITC, showing similar ratios for penetration over the time course to those of zilucoplan. Compared to zilucoplan and dextran 4 kDa, eculizumab biosimilar (labeled with AF488) whose concentrations in the lower and upper chambers were measured with the fluorescent intensity of AF488 displayed significantly lower ratios and slower kinetics over the entire time course. Its ratio at 24 h only reached approximately 0.12. Similarly, lower ratios and slower kinetics were also observed for FITC-conjugated dextran 150 kDa with a molecular weight close to that of an IgG antibody, including eculizumab biosimilar. These results are consistent with molecular weight being a major factor in determining the permeability (28, 29) and suggest improved tissue penetration of macrocyclic peptide inhibitors compared to antibody therapeutics.

## 4 Discussion

In this report, we demonstrate that zilucoplan binds C5 with high affinity and blocks the generation of anaphylatoxin C5a and cytolytic MAC by C5 convertases. Importantly, zilucoplan is also able to prevent the activation of clinical C5 R885C/H variants and thus has the potential to benefit patients who carry these polymorphisms and do not respond to treatment with eculizumab (16). The ability of zilucoplan to dissociate stable C5b6 complex and to inhibit a non-canonical pathway of TCC activation mediated such as by plasmin suggests a novel mechanism specific to zilucoplan by competing with C6 for the binding of C5b. Further, with a molecular weight 40-fold lower than that of IgG antibodies, zilucoplan displayed faster and greater penetration in an *in vitro* reconstituted basement membrane model, which may have implications for improved tissue penetration and biodistribution *in vivo*. This may partially explain the rapid onset of clinical benefit observed with zilucoplan during clinical studies in patients with generalized myasthenia gravis (14), a disease where the complement activation occurs at the neuromuscular junction (30).

There appear to be multiple interfaces on C5 that are crucial to its docking to C5 convertases via binding to C3b, as demonstrated by the fact that different potent C5 inhibitors bind to different locations on the C5 protein (21, 23, 31). Based on the cobra venom factor (CVF)-generated C5 convertase docking model and the structural homology between CVF and C3b, it has been proposed that the C5 MG4, MG5, and MG7 domains participate in interactions with human C5 convertases (22). Eculizumab with its epitope on C5 MG7 sterically clashes with C5 convertases (23). Tick protein CirpT with its epitope on C5 MG4 and MG5 inhibits the binding of C3b and C5, therefore blocking C5 cleavage and the MAC formation (21). However, C5 inhibitors that bind distinctive C5 domains distal from MG4, MG5, or MG7 were reported to inhibit C5 activation (19, 32). Zilucoplan represents a new type of C5 inhibitor with the binding site on the C5d/TED yet efficiently protecting C5 from being cleaved by C5 convertases.

Nishimura et al. first identified the C5 polymorphism R885H in a subset (~3.5%) of Japanese PNH patients who responded poorly to eculizumab treatment and demonstrated that the mutation led to the loss of binding, and therefore loss of efficacy, of eculizumab (16). The R885H mutation was also identified among healthy Japanese people with similar prevalence. Unlike eculizumab, which binds to R885 (23), zilucoplan binds to the distant C5d/TED of C5 and retains the full capacity to bind C5 R885C/H variants and to block their activation (**Figure 2**). Therefore, zilucoplan is likely to inhibit C5 activation in patients with such C5 polymorphisms.

The terminal complement pathway is initiated by the cleavage of C5 to C5a and C5b, involving significant conformational changes of C5 including a large rotation of the relatively rigid C5d/TED (25). Metastable C5b is a short-lived intermediate (half-life ~3 min) that is prone to form aggregates unless trapped by C6 to form a stable C5b6 complex (24). While it is challenging to directly measure ligand binding to nascent C5b, the dissociation of C5b6 and the inhibition of plasmin-mediated RBC lysis without blocking C5 cleavage by zilucoplan suggest that, by binding to the C5 TED moiety within C5b, zilucoplan inhibits the association of C5b with C6. This provides a second mechanism by which zilucoplan can prevent the assembly of MAC. To our best knowledge, zilucoplan is the only C5 inhibitor with a demonstrated ability to disrupt the stable C5b6 complex. Taken together, these data support a dual mechanism of action by which zilucoplan inhibits the activation of the terminal complement cascade via binding C5, including wild-type C5 and R885 variants, to prevent C5 convertase-mediated C5 cleavage into C5a and C5b, and additionally through remaining on the C5 TED moiety to hinder the formation of C5b6, efficiently blocking MAC formation that is central to targeting diseases such as acetylcholine receptor autoantibody-positive myasthenia gravis (33).

Designed with a smaller size and lower molecular weight than antibodies, a macrocyclic peptide may be able to penetrate tissues more effectively (28, 29). This was demonstrated in our *in vitro* permeability assay on a reconstituted basement membrane where zilucoplan showed greater permeability with faster kinetics than eculizumab biosimilar. The results in this *in vitro* model may translate to improved penetration and biodistribution *in vivo*, particularly in areas similar to the basement membrane model, such as the blood–nerve barrier, which might account for the rapid onset of action of zilucoplan seen in clinical studies in patients with AChR autoantibody-positive generalized myasthenia gravis (gMG) where AChR antibody-mediated complement activation occurs at the postsynaptic membrane of the neuromuscular junction.

In summary, the data presented here provide insights into a novel dual mechanism of action for zilucoplan as a potent inhibitor of terminal complement activation. Importantly, zilucoplan demonstrated full prevention of the activation of clinical C5 R885 variants that do not respond well to eculizumab treatment. They support the potential of zilucoplan as a next-generation C5 complement inhibitor to provide clinical benefits to patients with diseases driven by dysregulated activation of the terminal complement pathway.

## Data availability statement

The datasets presented in this article are not readily available because data from non-clinical studies is outside of UCB’s data sharing policy and is unavailable for sharing. Requests to access the datasets should be directed to Guo-Qing Tang, GuoQing.Tang@ucb.com.

## Author contributions

Assay and data analysis: KD, YT, MH, RV, G-QT, NZ, CB, J-GW, EP, BC, and ZM. Experiment design: DV, G-QT, YT, KD, MH, RV, NZ, ZM, J-GW, BC, and AR. Initial draft: G-QT, DV, and PD. Leadership: CS, AR, and PD. Editing: G-QT, PD, DV, CS, AR, J-GW, YT, KD, MH, RV, ZM, and EP. All authors contributed to the article and approved the submitted version.
