# Corrigendum: Zilucoplan, a macrocyclic peptide inhibitor of human complement component 5, uses a dual mode of action to prevent terminal complement pathway activation

**DOI:** 10.3389/fimmu.2023.1282155

**Published:** 2023-09-25

**Authors:** Guo-Qing Tang, Yalan Tang, Ketki Dhamnaskar, Michelle D. Hoarty, Rohit Vyasamneni, Douangsone D. Vadysirisack, Zhong Ma, Nanqun Zhu, Jian-Guo Wang, Charlie Bu, Bestine Cong, Elizabeth Palmer, Petra W. Duda, Camil Sayegh, Alonso Ricardo

**Affiliations:** ^1^UCB Pharma, Cambridge, MA, United States; ^2^Ra Pharmaceuticals, Cambridge, MA, United States; ^3^UCB Pharma/Ra Pharmaceuticals, Cambridge, MA, United States

**Keywords:** complement activation, C5 cleavage, C5 R885 variants, C5b6, MAC formation, macrocyclic peptide inhibitor, RBC hemolysis, permeability

In the published article, there were errors in **Figure 3** as published. In part A, the binding site is the “zilucoplan binding site”, rather than the “eculizumab binding site”. In part D, “nonspecific proteinases” should be “nonspecific proteases”. The corrected **Figure 3** and its caption appear below.

**Figure 3:**
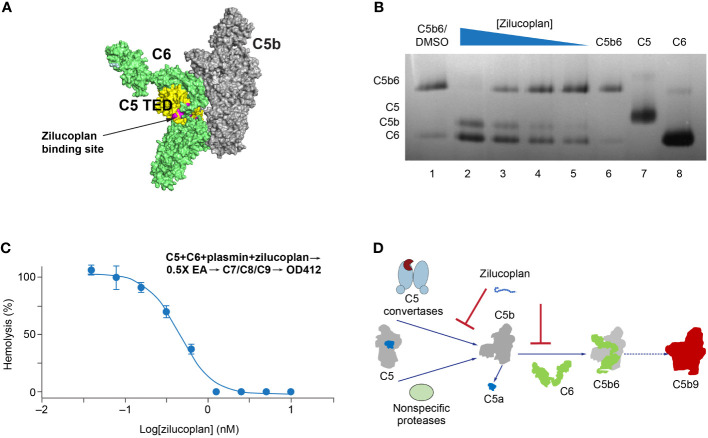
Zilucoplan destabilizes C5b6 and inhibits C5b6-mediated hemolysis lysis. **(A)** Schematic depiction (using PyMOL 2.4.0 based on PDB 4a5w) of the C5b6 complex. C5b is in gray except for the C5 TED in yellow, proposed binding sites for zilucoplan peptidyl portion based on a peptide analog (unpublished data) in pink, and C6 in lime. **(B)** Native gel analysis of the C5b6 complex in the presence and/or absence of zilucoplan. Lanes 1 and 6: C5b6 incubated at 37°C/h with (Lane 1) or without (Lane 6) 1% DMSO. Lanes 2–6: C5b6 after incubation with various concentrations of zilucoplan (in 1% DMSO) at 37°C for 1 h prior to gel loading. Lanes 7 and 8: C5 and C6 alone. **(C)** Zilucoplan inhibited the plasmin-mediated lysis of EA. Plasmin without or with increasing concentrations of zilucoplan was added to a mixture of C5 and C6 and incubated for 1 h. The plasmin reaction solution was then added to EA before mixing with purified C7, C8, and C9 to proceed with the lysis. More details are described in the *Materials and Methods*. Note that no sera were used to exclude any contribution by C3/C5 convertases. **(D)** A schematic diagram of the dual mechanism of action for zilucoplan: zilucoplan binds C5 (gray/dark blue) to prevent C5 cleavage into C5a (dark blue) and C5b (gray) by C5 convertases (light blue) and remains on C5b to prevent the formation of C5b6 (C6 in green) mediated by nonspecific proteases, both contributing to the blockade of MAC formation (red). TED, thioester-like domain; DMSO, dimethyl sulfoxide.

In the published article, there was an error. The **Introduction** incorrectly states that zilucoplan is composed of a 15-amino acid macrocyclic peptide including three unnatural amino acids. Zilucoplan is composed of a 15-amino acid macrocyclic peptide including four unnatural amino acids.

A correction has been made to **Introduction**, paragraph 4. This sentence previously stated:

“It is composed of a 15-amino acid macrocyclic peptide, including three unnatural amino acids, designed to inhibit TCC activation”

The corrected sentence appears below:

“It is composed of a 15-amino acid macrocyclic peptide, including four unnatural amino acids, designed to inhibit TCC activation”

The authors apologize for these errors and state that these do not change the scientific conclusions of the article in any way. The original article has been updated.

