# Supplementary material for: Zilucoplan, a macrocyclic peptide inhibitor of human complement component 5, uses a dual mode of action to prevent terminal complement pathway activation
Source: Front Immunol. 2023 Aug 9;14:1213920. doi: 10.3389/fimmu.2023.1213920 (PMC10446491; doi:10.3389/fimmu.2023.1213920)
Supplement: Supplementary file 1 [file DataSheet_1.docx]

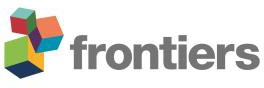


***Supplementary Material***

## Supplementary Materials and Methods

- 1. **MAC (C5b-9) and C3b deposition on HUVEC induced by anti-CD59 antibody**

Human umbilical vein endothelial cells (HUVEC, Lonza Walkersville, Cat# CC-2519) were cultured in MCDB-131 Complete Medium (Vec Technologies, Cat# MCDB131C) as described previously. Normal human serum (Cat# NHS), human C9-depleted serum (Cat# A326) and Human C9 protein (Cat# A126) were purchased from Complement Technologies (Tyler, Texas). Human C9 protein was conjugated with Alexa Fluor 647 dye using a labeling kit (Invitrogen Cat# A20186).

HUVECs (Passage#4, 30,000 cells/well) were seeded in a 96-well plate with a black and clear bottom (Corning Cat# 3603) and grown for 4 days to reach 100% confluency. HUVEC monolayers were washed twice with MEM medium supplemented with 0.1% bovine serum albumin (BSA) (MEM/BSA) and incubated with 4 µg/mL of mouse IgG2a isotype control (Biolegend, Cat# 401502) or anti-CD59 antibody (Clone MEM-43, Invitrogen, Cat# MA1-82206) for 30 minutes at 37°C in a CO_2_ incubator.

Normal human serum or human C9-depleted serum (supplemented with 60 µg/mL AF647- conjugated C9 protein) was preincubated with DMSO control or zilucoplan (final concentration 1.0 µM) for 15 minutes at room temperature. After washing 3 times with MEM/BSA buffer, HUVEC monolayers were incubated with 100 µL of 15% NHS or C9-depleted serum (+AF647-C9) for 15 minutes at 37°C before fixation with 4% paraformaldehyde (Sigma, Cat# 47608) solution.

Complement C3b was stained with H11 antibody (Invitrogen, Cat# MA1-82934) and

AF555-conjugated goat anti-mouse IgG1 (Invitrogen, Cat# A21127). CD31/PECAM-1 was stained with a rabbit polyclonal antibody (Invitrogen Cat# PA5-16301) and AF488-conjugated goat-anti- rabbit IgG second antibody (Jackson Immunoresearch, Cat# 111-545-046). Nucleus was stained with Hoechst 33342 solution (Invitrogen Cat# H3570). Images from each well were collected in a Monta mode (2×2 or 3×3) using a Cytation 5 imager reader (Bio-Tek). Fluorescence intensity was shown as mean ± SD (n = 3–4 wells each group). Statistical significance was analyzed by ANOVA (Kruskal-Wallis test) using GraphPad Prism software (Version 9.2.0).

## Preparation of recombinant human C5 wild-type and variants

Recombinant human C5 wild-type (rhC5 wt) and variants with a single mutation at R885 (rhC5 R885C or R885H) were expressed transiently using the Expi293 cell line. Culture medium expressing target C5 protein was concentrated using tangential flow filtration to 100 mL and diluted with 200 mL of 20 mM HEPES pH 7.4 buffer. This was applied to a HiTrap Q HP column and the elution collected with a NaCl gradient. Fractions containing the target C5 protein were pooled and dialysis used for buffer exchange to 50 mM NaCl, 20 mM HEPES, pH 6.5. The mixture was applied to a second ion exchange column (HiTrap SP) and eluted with a NaCl segmental gradient; fractions containing target C5 protein were pooled and the buffer changed to 20 mM HEPES, pH 7.4, 100 mM NaCl. Aliquots were snap frozen and stored at −80°C. SDS-PAGE characterization of recombinant C5 wt and variants R885C and R885H are shown in Supplementary Figure 3.

## Permeability evaluation using reconstituted basement membrane

A working stock of zilucoplan of concentration 1 mg/mL was prepared in 1× PBS pH 7.4 buffer. Eculizumab biosimilar (Syd Labs) was dialyzed into 1×PBS (pH 7.4) and labeled with Alexa Fluor™ 488 NHS Ester (AF488) (ThermoFisher). Excess Alexa Fluor 488 was removed by 10kD Amicon Ultra-

0.5 Centrifugal Filter (MillporeSigma). The concentrations of eculizumab biosimilar and labeled AF488 were determined by absorbance measurement. The labeling ratio for AF488 to protein 0.7 was determined to be 0.7. Transwell plates and Matrigel were purchased from Corning Life Sciences. All high-performance liquid chromatography (HPLC) solvents were of HPLC-mass spectrometry (MS) grade and were obtained from ThermoFisher.

A 2 mg/mL Matrigel solution was prepared using ice-cold water with gentle mixing and used to coat the transwell membrane. The matrigel was allowed to dry at 37^o^C for 24 hours, and stored at 4^o^C, wrapped in plastic film prior to use. 200 µL of PBS solutions of zilucoplan, AF488-labeled eculizumab biosimilar, FITC-labeled dextran 4 kD, or FITC-dextran 150 kD (all in 3 µM) were transferred to each upper transwell chamber. Meanwhile, 900 µL PBS buffer was transferred to each lower transwell chamber. The transwells were then cultured at 37^o^C, and aliquots of samples were collected at 1, 2, 4, 8, 12, and 24 hours post incubation. For eculizumab biosimilar or FITC-dextran or PBS blank,

100 µL of incubation sample was collected from both upper and lower chambers at each time point and transferred to the micro plate for the measurement using fluorescent assay. For zilucoplan, at each time point an aliquot of 200 or 900 µL of acetonitrile containing 2 µM of glyburide as the internal standard was added to the upper or lower chamber, respectively, and mixed well. 200 µL of the resulting mixture was transferred to centrifuge tubes. Following vortex and centrifugation, the resulting supernatant was transferred to HPLC vials for HPLC-MS analysis. The assay was conducted in duplicate in each of two plates for each test article at each time point. Fluorescence intensity of AF488 or FITC was measured on a Tecan Spark microplate reader (excitation at 490 nm, bandwidth of 5 nm, and emission at 520 nm, bandwidth of 15 nm). A Q-exactive mass spectrometer (ThermoFisher), coupled with an UltiMate 3000 HPLC 1200 system (ThermoFisher) with a Kinetex C18 column, was used for HPLC-MS analysis of zilucoplan. The Q-exactive mass spectrometer was interfaced with an electrospray ionization source and operated in positive mode, with resolving power of 70,000 for MS data collection.

## 2. Supplementary Figures

**Supplementary Figure 1. Zilucoplan blocks the deposition of MAC but not C3b fragments on HUVEC induced by anti-CD59 Ab.** C3b deposition was detected using AF555 labeled anti-C3b mAb (H11). MAC deposition was detected by AF647-C9 protein in C9-depleted human sera supplemented by AF647-C9. Endothelial cell surface marker CD31 was stained using a rabbit polyclonal antibody and AF488 conjugated goat anti-rabbit IgG. Fluorescence intensity was shown as mean ± SD (n=3 or 4 each group). Statistical significance was analyzed by ANOVA (Kruskal-Wallis test) using GraphPad software (Version 9.2.0) (Ab, antibody; DMSO, dimethyl sulfoxide).


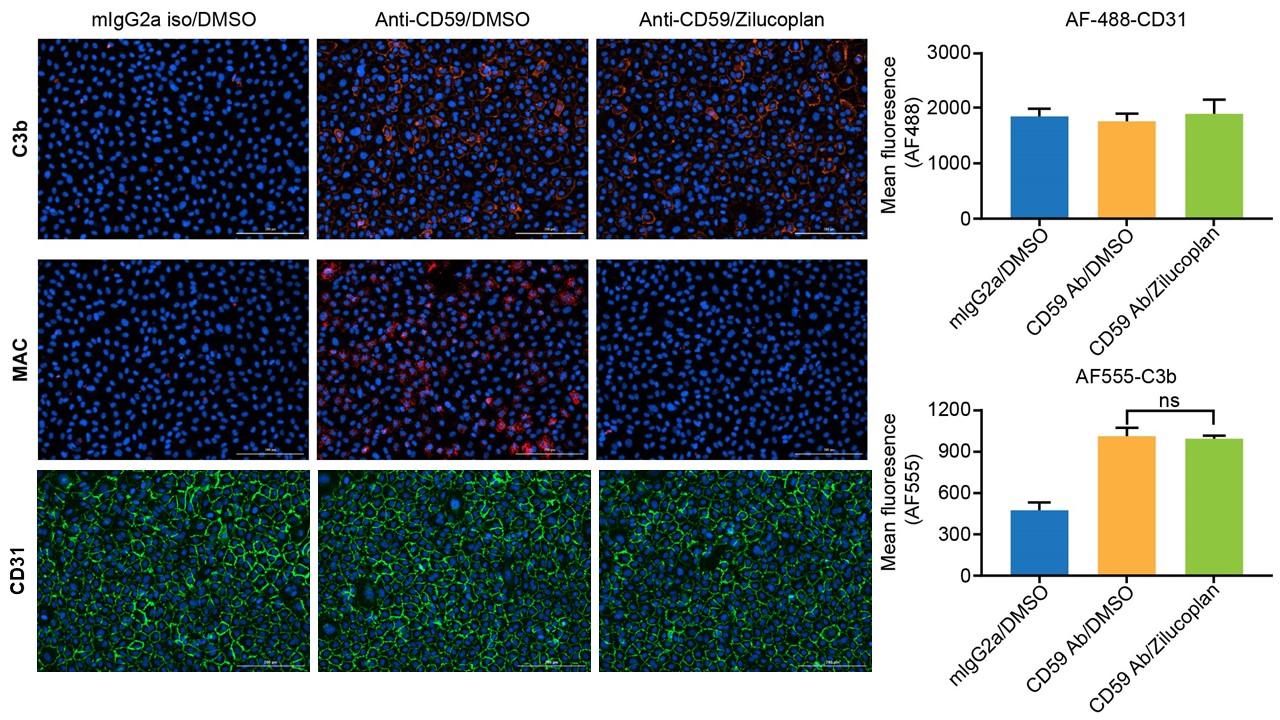


**Supplementary Figure 2. Determination of CH50 of the hemolysis by C5 from different sources.** Wild type C5 purified from human sera (hC5 compTech) (Complement Technologies), recombinant C5 wild type (rhC5 WT), recombinant C5 variant R885C (rhC5 R885C), and recombinant C5 variant R885H (rhC5 R885H). Various concentrations of protein C5 were added to 1.5% C5-depleted human sera followed by incubation with EA (0.5×) in GVB++ buffer. All data were from 2 plates (duplicate in each plate).


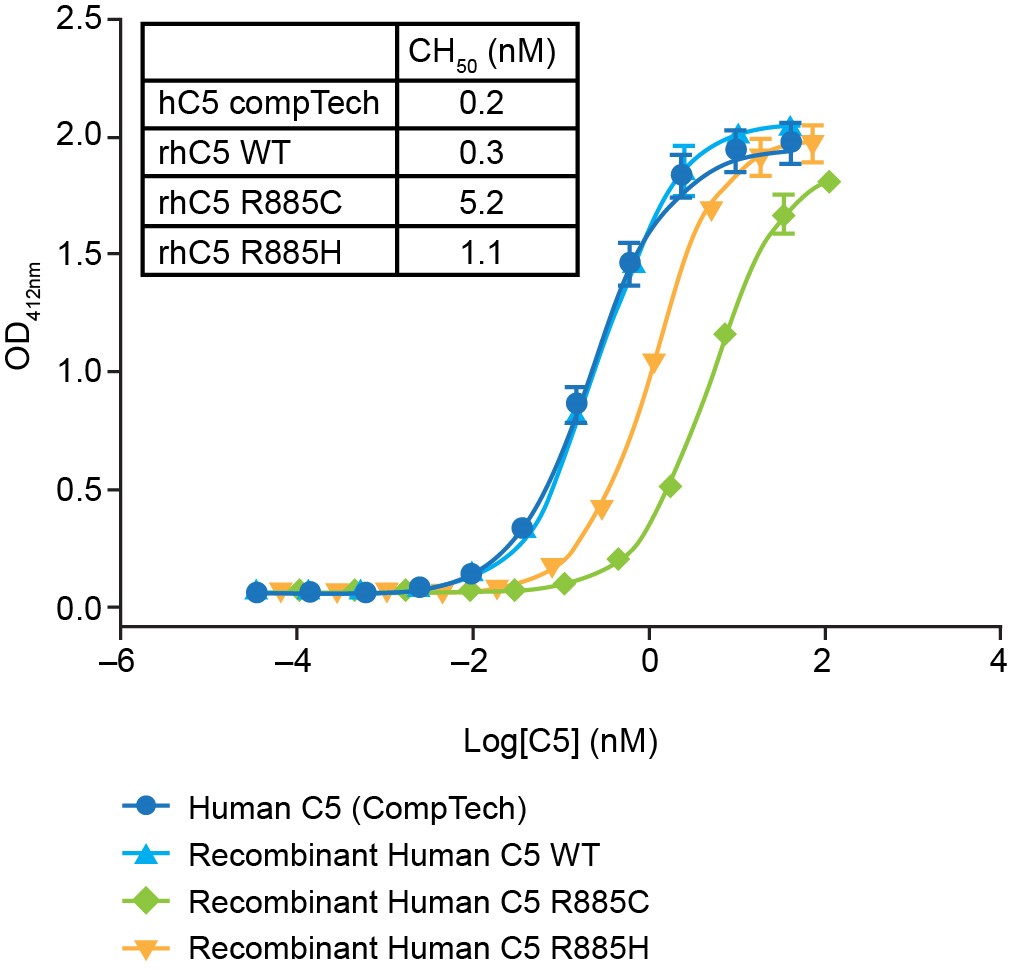


**Supplementary Figure 3. Coomassie staining and western blotting.**

# Coomassie staining

**C5 rhC5**

**rhC5**

**rhC5**

**C5 rhC5**

**rhC5**

**rhC5**

**CompTech**


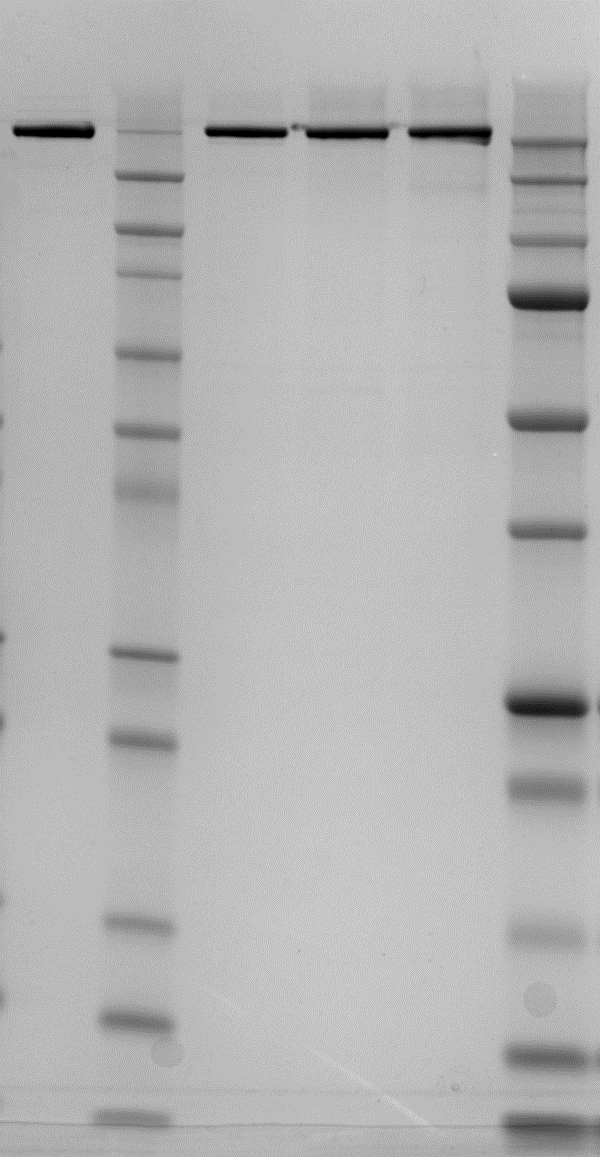


Marker

(kDa)

Marker

(kDa)

260

160

110

80

100

75

60

50

50

40

37

30

25

20

20

15

15

10

10

3.5

5

**WT R885H R885C**

**CompTech**

**WT R885H R885C**

| 250 | 250 | 260 |
| --- | --- | --- |
| 150 | 150 | 160 |

**Line**


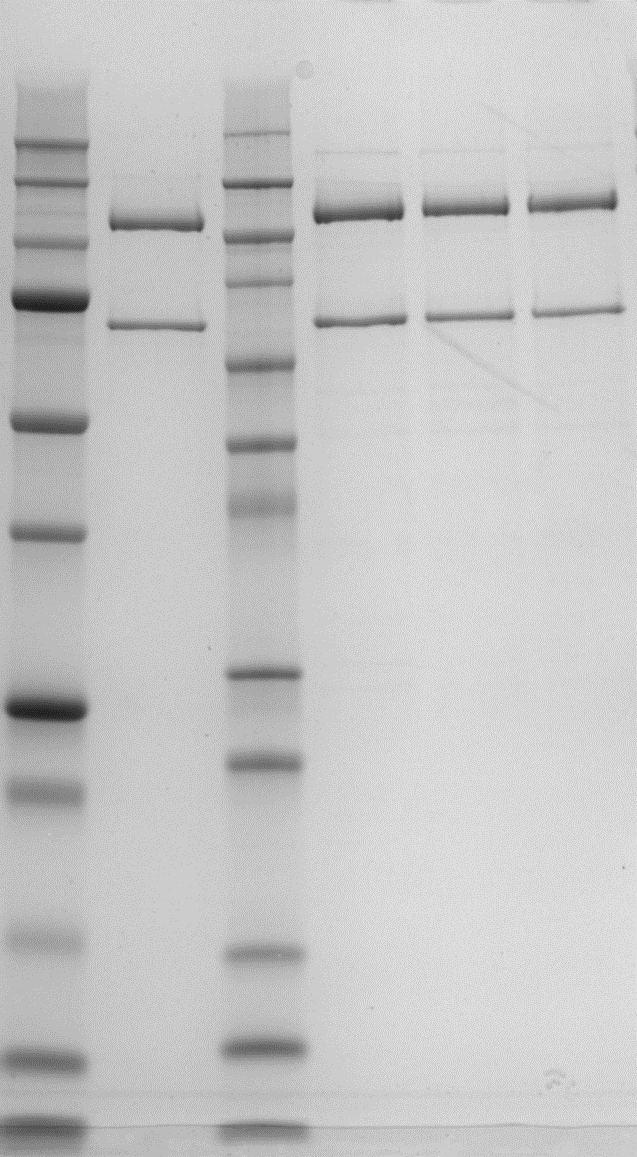


Marker

(kDa)

Marker

(kDa)

100

75

110

80

60

50

50

40

37

30

25

20

20

15

15

10

10

5

3.5

**1 2 3 4 5 6**

**Non-reducing condition**

**Line 1 2 3 4 5** **6**

**Reducing condition**

**Non-reducing condition**

Line:

1. 1 µg human C5 CompTech #A120 Lot: #23f
2. annotated protein marker, ThermoFisher #LC5800 Novex™ Sharp Pre‐stained Protein Standards
3. 1 µg recombinant C5 wild type (rhC5 wt)
4. 1 µg recombinant C5 R885H (rhC5 R885H)
5. 1 µg recombinant C5 R885C (rhC5 R885C)
6. Annotated protein marker, BioRad #161‐0377 Precision Plus Protein™ Dual Xtra Standards

**Reducing condition**

Line:

1. Annotated protein marker, BioRad #161‐0377 Precision Plus Protein™ Dual Xtra Standards
2. 1 µg human C5 CompTech #A120 Lot: #23f
3. annotated protein marker, ThermoFisher #LC5800 Novex™ Sharp Pre‐stained Protein Standards
4. 1 µg recombinant C5 wild type (rhC5 wt)
5. 1 µg recombinant C5 R885H (rhC5 R885H)
6. 1 µg recombinant C5 R885C (rhC5 R885C)

# Western blotting

**C5 rhC5 rhC5 CompTech WT R885H**

**rhC5 R885C**

Marker (kDa)

Marker **C5**

(kDa) **CompTech**

**rhC5 rhC5**

**WT R885H**

**rhC5 R885C**


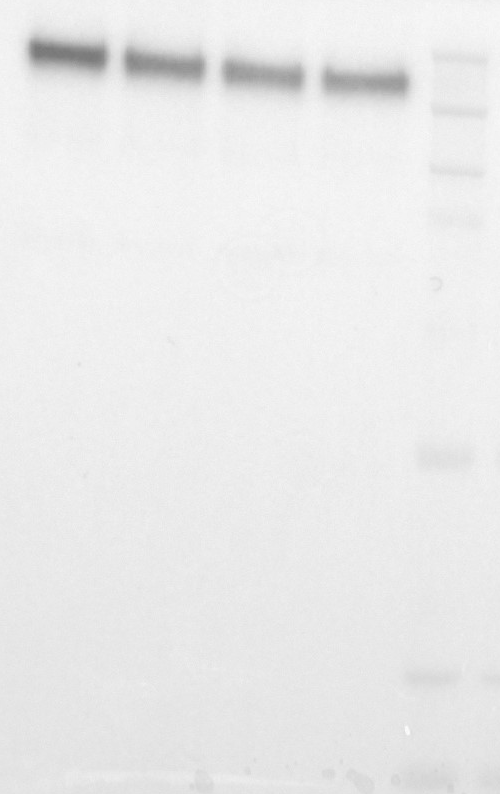


250

150

100

75

50

37

25

20


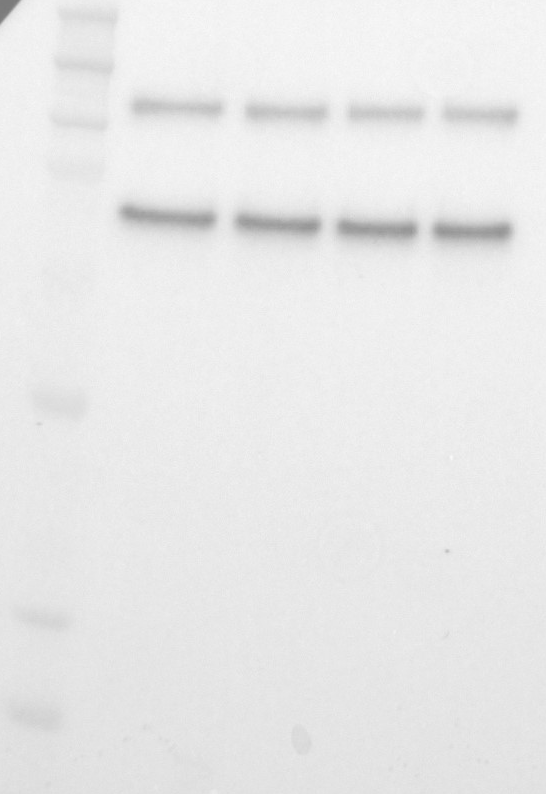


250

150

100

75

50

37

25

20

**Line**

**1 2 3 4 5**

**Non-reducing condition**

**Line** **1**

**2 3 4 5**

**Reducing condition**

**Non-reducing condition**

Line:

- 1. 50 ng human C5 CompTech #A120 Lot: #23f
  2. 50 ng recombinant C5 wild type (rhC5 wt)
  3. 50 ng recombinant C5 R885H (rhC5 R885H)
  4. 50 ng recombinant C5 R885C (rhC5 R885C)
  5. Annotated protein marker, BioRad #161‐0377 Precision Plus Protein™ Dual Xtra Standards

**Reducing condition**

Line:

1. Annotated protein marker, BioRad #161‐0377 Precision Plus Protein™ Dual Xtra Standards
2. 50 ng human C5 CompTech #A120 Lot: #23f
3. 50 ng recombinant C5 wild type (rhC5 wt)
4. 50 ng recombinant C5 R885H (rhC5 R885H)
5. 50 ng recombinant C5 R885C (rhC5 R885C)

Primary antibody: Goat Anti‐Human C5 (CompTech #A220, Lot 2: 2e) 1:8000 dilution with 5% BSA, 1X Tris-Buffered Saline, 0.1% Tween® 20 Detergent (TBST).

Secondary antibody: Rabbit Anti‐Goat IgG (H+L) (Thermo Fisher, SA5-10314), Lot: WI3393022), 1:10,000 dilution with 5% BSA 1x TBST.
